# The First Report of a 290-bp Deletion in *β*-*Globin* Gene in the South of Iran

**DOI:** 10.18869/acadpub.ibj.21.2.126

**Published:** 2017-03

**Authors:** Mohammad Hamid, Ladan Dawoody Nejad, Gholamreza Shariati, Hamid Galehdari, Alihossein Saberi, Marziye Mohammadi-Anaei

**Affiliations:** 1 Department of Molecular Medicine, Biotechnology Research Center, Pasteur Institute of Iran, Tehran, Iran; 2 Narges Medical Genetics and Prenatal Diagnosis Laboratory, No. 18, East Mihan Ave., Kianpars, Ahvaz, Iran; 3 Department of Medical Genetic, Faculty of Medicine, Ahvaz Jundishapur University of Medical Sciences, Ahvaz, Iran

**Keywords:** β-thalassemia, *β*-*globin* gene mutation, Iran, Multiplex ligation-dependent probe amplification

## Abstract

**Background:**

β-thalassemia is one of the most widespread diseases in the world, including Iran. In this study, we reported, for the first time, a 290-bp *β*-*globin* gene deletion in the south of Iran.

**Methods:**

Four individuals from three unrelated families with Arabic ethnic background were studied in Khuzestan Province. Red blood cell indices and hemoglobin analysis were carried out according to the standard methods. Genomic DNA was obtained from peripheral blood cells by salting out procedures. *β*-*globin* gene amplification, multiplex ligation-dependent probe amplification (MLPA), and DNA sequencing were performed.

**Results:**

The PCR followed by sequencing and MLPA test of the *β-globin* gene confirmed the presence of a 290-bp deletion in the heterozygous form, along with -88C>A mutation. All the individuals had elevated hemoglobin A_2_ and normal fetal hemoglobin levels.

**Conclusions:**

This mutation causes β^0^-thalassemia and can be highly useful for prenatal diagnosis in compound heterozygous condition with different *β-globin* gene mutations.

## INTRODUCTION

β -thalassemia is one of the most frequent genetic disorders in Iran with a great mutational diversity. More than 280 mutations have been identified in association with β-thalassemia in the country. Mutations are mostly single base substitutions, and in some cases, they may cause deletions or insertions of different regions in a gene[1,2]. *β-globin* gene deletions, especially those in promoter regions, are usually associated with the high levels of hemoglobin A_2_ (HbA_2_) in heterozygous individuals. In addition, the high levels of fetal hemoglobin (HbF) are detected because of *δβ-globin* gene deletions. However, large deletions of *δβ-* and *γ-globin* genes are observed among some carriers with normal levels of HbF. Since most deletions may be missed by DNA sequencing, the identification of deletions in *β-globin* gene by other means is of great important[3,4]. In the current study, we detected a 290-bp deletion in four individuals from three unrelated families with Arabic ethnic background in Khuzestan Province, south of Iran.

## MATERIALS AND METHODS

The present investigation is a part of a national program for the prevention of thalassemia. In total, four individuals who referred to the Narges Prenatal Diagnostics and Medical Genetics Laboratory (Ahvaz, Iran) during three years participated in this study. The analysis of red blood cell indices and Hb analysis were carried out according to the standard methods. Following a written informed consent from the subjects, some molecular studies were conducted on the genomic DNA isolated from peripheral blood cells using a salting out procedure[5]. To identify α-thalassemia genotype, the common Mediterranean *α-globin* gene deletions were investigated by Gap-PCR as described elsewhere[6]. *β-globin* gene was amplified and directly sequenced by the chain termination method[7] on the ABI PRISM 3130 Genetic Analyzer (Applied Biosystems, Foster City, CA, USA).

For detection of deletions, multiplex ligation-dependent probe amplification (MLPA) assay was performed using the SALSA MLPA kit P102 HBB (MRC-Holland, Amsterdam, Netherlands). Then amplified fragments were separated by capillary electrophoresis on the ABI PRISM 3130 Genetic Analyzer (Applied Biosystems, Foster city, CA, USA) and analyzed by GeneMarker software v.1.6 (Soft Genetics, State College, PA, USA).

## RESULTS

This study reported, for the first time, a 290-bp deletion (c.-176_92+25del) in *β-globin* gene in four individuals from three unrelated families with Arabic ethnic background in Khuzestan Province. All the individuals had elevated HbA_2_ and normal HbF levels. One of the individuals, offspring of K.B., was a 5-year-old girl, who inherited both defects (290-bp deletion/-88C>A mutation) from her parents. Physical examination of the patient indicated pallor, slight hepatosplenomegaly. The hematological and molecular data of the studied subjects are summarized in Table 1.

**Table 1:** The hematological and molecular data of the studied subjects

	S.M.	M.M.	K.B.	Offspring of K.B.
Age (year)	18	24	48	5
Gender	F	M	M	F
Hb (g/dl)	10.9	11.5	12.5	6.4
RBC (10^12^/L)	5.19	6.33	5.99	3.58
MCV(fl)	64.5	64.8	66.8	59.3
MCH (pg)	21	21.8	20.9	17.9
MCHC (%)	32.5	-	31.3	30.2
HbA (%)	94.6	92.9	-	94.8
HbF (%)	0.4	0.6	-	0.0
HbA_2_ (%)	5	6.5	-	5.2
β-genotype	290-bp deletion/N	290-bp deletion/N	290-bp deletion /N	290-bp deletion/-88 C>A
α-genotype	αα/αα	αα/αα	αα/αα	αα/αα
Origin	Arab	Arab	Arab	Arab

F, female; M, male; N, normal

**Fig. 1:**
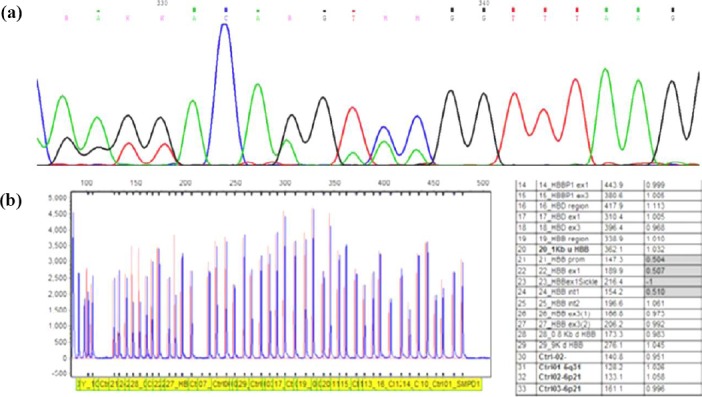
The chromatogram and histogram of multiplex ligation-dependent probe amplification (MLPA) dosage showing a 290-bp deletion on *β-globin* gene. (a) The sequence of a 290-bp deletion presenting in heterozygosity; (b) The histogram of MLPA showing a 290-bp deletion.

The 290-bp deletion was characterized by DNA sequencing and MLPA test. The mutation removed the region between positions -125 and +78 relative to the *β-globin* gene mRNA cap site. The MLPA results confirmed the deletion by probes ranging from prob 21 (Promoter) to prob 1 (HBB intron 1) (Fig. 1). Generally, no mutation or deletion was found in the *α-globin* genes of the studied individuals.

## DISCUSSION

The 290-bp deletion was first observed in a Turkish patient and later in many other patients[8-11]. In the present investigation, we reported, for the first time, a 290-bp deletion along with -88C>A mutation in the south of Iran.

The mutation removed the region between positions -125 and +78 relative to the *β-globin* gene mRNA cap site. According to a previous study[12], -88C>A mutation allows the β-locus control region to interact with the promoters of δ- and *γ-globin* genes by competition between fetal and adult globin genes, which result in HbA_2_ and HbF levels[12]. However, our samples with a 290-bp deletion were just associated with the increased levels of HbA_2_ and normal HbF. Some of the deleted elements in positions -125 and +78 are the CAC (−90), CAAT (−70) and TATA (−30) boxes. The absence of these elements is led to increased HbA_2_ levels without any effect on *γ-globin* genes, which is in contrast to the previous reports[8-10,13].

The detection of this β-thalassemia deletion in the promoter region, which is an available place for transcription factors, can be highly useful for prenatal diagnosis as the consanguineous and ethnic marriages in families compatible the control of the disease.
